# Effects of Araticum (*Annona crassiflora* Mart.) By‐Product on Intestinal Health Parameters in High‐Fat Diet‐Fed Mice

**DOI:** 10.1002/fsn3.72163

**Published:** 2026-07-29

**Authors:** Thamara Rosa de Souza, Fellipe Lopes de Oliveira, Sabrina Neves Casarotti, Suélem Aparecida de França Lemes, Eudes Thiago Pereira Avila, Amílcar Sabino Damazo, Lucas Amoroso Lopes de Carvalho, Daniel Guariz Pinheiro, Katia Sivieri, Maressa Caldeira Morzelle

**Affiliations:** ^1^ Faculty of Nutrition Federal University of Mato Grosso Cuiabá Mato Grosso Brazil; ^2^ Universidade Do Estado de São Paulo Araraquara São Paulo Brazil; ^3^ Faculty of Health Sciences Federal University of Rondonópolis Rondonópolis Mato Grosso Brazil; ^4^ Federal University of Mato Grosso, Chemical Institute Cuiabá Mato Grosso Brazil; ^5^ Faculty of Medicine Federal University of Mato Grosso Cuiabá Mato Grosso Brazil; ^6^ Department of Agricultural, Livestock and Environmental Biotechnology São Paulo State University (UNESP), School of Agricultural and Veterinary Sciences Jaboticabal São Paulo Brazil; ^7^ Graduate Program in Biotechnology in Regenerative Medicine and Medicinal Chemistry Food University of Araraquara (UNIARA) Araraquara São Paulo Brazil

**Keywords:** Brazilian fruits, dietary fiber, dysbiosis, intestinal health, prebiotics

## Abstract

Obesity is a major public health issue associated with metabolic disturbances and alteration in the gut microbiota. Studies indicate that bioactive compounds can modulate these processes, and fruit by‐products represent a promising source of such compounds. This study investigated the impact of araticum (*Annona crassiflora* Mart.) by‐product on intestinal microbiota composition and obesity biomarkers in mice fed a high‐fat diet. The by‐product was characterized for technological and proximate composition. Forty‐six Swiss mice were randomized into four groups: a control diet group, a high‐fat diet group, a high‐fat diet + araticum by‐product (200 mg kg‐1) group, and a high‐fat diet + araticum by‐product (400 mg kg‐1) group. After 30 days, murinometric, biochemical, and histological parameters and the composition of microbiota were assessed. The by‐product contained 8.04 g.100 g‐1 of moisture, 11.17 g.100 g‐1 of protein, 9.79 g.100 g‐1 of lipids, 53.29 g.100 g‐1 of total fiber (3.96 g.100 g‐1 soluble and 49.33 g.100 g‐1 insoluble), 2.97 g.100 g‐1 of ash, and 14.74 g.100 g‐1 of digestible carbohydrates. Araticum by‐product consumption did not affect weight gain or glucose tolerance but increased fecal moisture content and reduced pH. Regarding gut microbiota composition, the supplemented groups showed a trend toward a higher relative abundance of Firmicutes. In the group receiving the highest dose of araticum by‐product (400 mg kg^−1^), more intense intestinal PAS staining was observed, which may indicate a greater presence of PAS‐positive mucin‐containing structures. These preliminary findings suggest that the araticum by‐product may influence selected intestinal parameters, although its functional effects require confirmation in longer‐term and mechanistic studies.

## Graphical abstract

Araticum (*Annona crassiflora* Mart.) by‐product supplementation at 200 or 400 mg/kg for 30 days did not improve body weight, glucose tolerance, or obesity‐related metabolic markers in high‐fat diet‐fed mice. However, it increased fecal moisture and intestinal mucus, reduced fecal pH, and promoted changes in gut bacterial richness, indicating potential benefits for intestinal health.

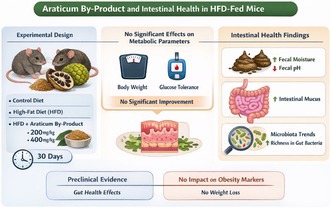

## 1 Introduction

Recent changes in the global population's lifestyle, particularly the introduction of diets rich in lipids and simple carbohydrates, are among the primary factors contributing to the increasing prevalence of obesity. Obesity is associated with various disorders, including cardiovascular disease, type 2 diabetes, hypertension, and certain types of cancer (Zhang et al. 2024).

Obese individuals exhibit alterations in the intestinal microbiota, which has prompted numerous studies to explore this relationship (Makki et al. 2018; Pan and Jiao 2024; Sun et al. 2022). Dysbiosis is characterized by changes in both the composition and function of the intestinal microbiota, leading to local inflammation, disruption of tight junctions in enterocytes, and increased intestinal barrier permeability (Makki et al. 2018). These conditions facilitate the growth of pathogenic bacteria and their toxic by‐products, such as lipopolysaccharides (LPS). When LPS enters the bloodstream, it is associated with metabolic endotoxemia, systemic inflammation, peripheral insulin resistance, and hepatic steatosis, conditions commonly linked to obesity (Cani et al. 2008; Winer et al. 2016).

Among the strategies recommended to restore the balance of the intestinal microbiota are increased consumption of dietary fiber and prebiotics, as well as the inclusion of probiotics and/or symbiotic combinations in the diet (Gasmi et al. 2023; Gibson et al. 2017; Rau et al. 2024; Yadav et al. 2022). Evidence suggests that fruit by‐products can serve as alternative sources of biologically active compounds with the potential to mitigate obesity, insulin resistance, and inflammatory diseases, while also interacting with beneficial microorganisms in the microbiota to produce short‐chain fatty acids, which provide additional benefits to the host (Batista et al. 2018; Lima et al. 2023).

Studies exploring the prebiotic potential of fruit by‐products have mainly concentrated on conventionally consumed species such as guava, orange, grape, acerola, cashew, passion fruit, and jaboticaba (Batista et al. 2018; Casarotti et al. 2018; Gil‐Sánchez et al. 2018). In contrast, unconventional fruits from the Brazilian Cerrado, such as araticum, remain largely underexplored regarding their prebiotic properties.

Araticum (*Annona crassiflora* Mart.) is a fruit native to the Brazilian cerrado, commonly known as marolo, bruto, or paña. The pulp is consumed fresh or in juices and jellies by local communities, while the peel and seeds are typically discarded or used as animal feed. These by‐products, composed of the peel and seeds, are rich in dietary fiber, which may have potential applications in the food industry (Arruda et al. 2023; Oliveira et al. 2022; Roesler et al. 2007). Incorporating fruit by‐products aligns with the principles of the Sustainable Development Goals (SDGs), promoting responsible consumption and production patterns to reduce food waste and minimize the environmental impact throughout the production chain (United Nations 2024).

Given this context, the present study investigated the effects of the araticum by‐product on intestinal microbiota composition and obesity‐related biomarkers in mice fed a high‐fat diet. The rationale for selecting this by‐product lies in the increasing interest in valorizing agro‐industrial residues as natural sources of bioactive compounds and dietary fibers with potential health‐promoting effects. Araticum, a native fruit of the Brazilian Cerrado, is particularly rich in phenolic compounds, complex carbohydrates, and fibers that may contribute to metabolic regulation and intestinal homeostasis. By integrating nutritional, biochemical, and histological analyses, this study aimed to elucidate the physiological responses associated with araticum by‐product consumption under high‐fat dietary conditions. Beyond its nutritional relevance, the investigation highlights the environmental and economic benefits of utilizing fruit‐processing residues, supporting the principles of sustainability and circular bioeconomy. The findings may provide a scientific basis for future applications of araticum by‐products in the development of functional foods targeting metabolic health.

## 2 Materials and Methods

### 2.1 Araticum By‐Product

The by‐product obtained from the seeds and peel of araticum (*Annona crassiflora* Mart.) was prepared according to the methodology of De Oliveira et al. (2023). The fresh araticum fruits (80 kg) were harvested in the city of Januária, Minas Gerais—Brazil. The fruits were cleaned and pulped manually. The by‐product was dried to constant weight in an oven with air circulation at 60°C (SOLAB, model SL‐102, Piracicaba, SP, Brazil), crushed (Oster, model Osterizer Blender), and passed through a sieve (Granutest) with an opening of 0.42 mm. The by‐product was portioned (50 g), vacuum‐packed, and irradiated using a dose of 5 kGy.

### 2.2 Chemical Characterization of Araticum By‐Product

The proximate composition of the by‐product was determined according to AOAC (1995): moisture content (direct drying at 105°C, method 925.10), ash (incineration in a muffle furnace at 550°C, method 923.03), total protein (Kjeldahl method, method 920.39), lipids (Soxhlet), and soluble and insoluble fibers (method 991.43). Total fiber was calculated as the sum of the soluble and insoluble fractions, and digestible carbohydrates were calculated by difference.

The water absorption index (WIA) and water solubility (WSS) were determined according to the methodology described by Anderson et al. (1970) and Becker et al. (2014). The oil absorption index (OAI) was calculated according to the methodology of Anderson et al. (1970) and Becker et al. (2014), with water replaced by oil. The sample was analyzed in quadruplicate.

### 2.3 Animals

Male Swiss mice (*n* = 46) of 6–8 weeks of age (average weight of 30–40 g) were maintained in individually ventilated cages (Alesco, Monte Mor, SP, Brasil), with food and water ad libitum. Room temperature was 22°C to 26°C and humidity was 55%.

After the adaptation period (7 days), the animals were randomized into two groups: CTRL—control group that received a normolipid diet (*n* = 12), and HFD—group that received a high‐fat diet (*n* = 34). After 15 days of consuming the diet, the animals in the HFD group were divided into three experimental groups: HFD (hyperlipidic group that did not receive the araticum by‐product, *n* = 10); HFD‐A200 (hyperlipidic group treated with araticum by‐product at 200 mg kg‐1, *n* = 12); HFD‐A400 (hyperlipidic group treated with araticum by‐product at 400 mg kg‐1, *n* = 12).

All efforts were made to reduce the number of animals and their suffering. The experimental proceedings were performed according to the ARRIVE guidelines (Animal Research: Reporting in Vivo Experiments). The experimental protocols were approved by the Animal Use Ethics Committee from Federal University of Mato Grosso under number 23108.091554/2021‐48.

### 2.4 Induction of Dysbiosis With High Fat Diet and Treatment With Araticum By‐Product

The high‐fat diet was prepared according to Magalhães et al. (2019) in the following proportions: 66.5% rodent feed (Nuvilab‐CRN1), 13.5% pork lard, and 20% sugar. The rodent feed was ground in a processor (Oster, model Osterizer Blender) and then passed through a sieve (Granutest) with an opening of 0.48 mm. The feed was shaped into pellets and then dried in an oven (SOLAB, model SL‐102, Piracicaba, SP, Brazil) with air circulation at 60°C for 48 h.

The commercial Nuvilab‐CRN1 diet provides 350 Kcal (1470 KJ), while the hyperlipidic diet has 481 Kcal/100 g (2019 KJ). The Nuvilab‐CRN1 diet contains, per 100 g, 74 g of carbohydrates, 22 g of protein, and 4 g of total fat, whereas the hyperlipidic diet has 69 g of carbohydrates, 15 g of protein, and 16 g of fat. The latter also contains 5.3 g of saturated fat, while the former contains none. Iron and zinc are higher in Nuvilab‐CRN1, with 50 mg and 60 mg, respectively.

Initially, a solubility test (unpublished data) was carried out on the araticum by‐product with water and oil. The araticum by‐product has low solubility in water, so oil was chosen to administer the by‐product to the animals. The dosage of araticum by‐product was chosen based on the study by Batista et al. (2018), who investigated the effects of fruit by‐products on rats with dyslipidemia. The CTRL and HFD groups received sunflower oil (vehicle), and the HFD‐A200 and HFD‐A400 groups received the araticum by‐product solution and the vehicle, administered by orogastric gavage once a day for 30 days.

### 2.5 Feeding Behavior, Biochemical Tests, and Murine Parameters

Food intake was monitored daily. The offered feed was weighed using a scale (Marconi, model BL3200H), and after 24 h, the remaining feed in the cage was re‐weighed. Energy intake (KJ/day) was calculated based on the energy value of the diet, the vehicle, and any by‐products provided to the mice.

At the end of the experiment, the animals were fasted overnight (12 h) and their blood glucose was checked in the peripheral vein using a glucometer (Accu‐Chek Active). For the biochemical tests, the animals were anesthetized with Ketamine: Xylazine (110:80 mg/kg i.p.). Blood samples were then collected by cardiac puncture in tubes with and without anticoagulant to collect serum and plasma and analyzed for triglycerides, alanine aminotransferase (ALT), aspartate aminotransferase (AST), urea, and creatinine. All the biochemical tests were carried out using an enzymatic colorimetric system according to the manufacturer's guidelines, Bioclin. Subsequently, euthanasia was carried out by cervical dislocation.

To observe changes in murine parameters, weight was monitored daily and chest circumference, abdominal circumference, and naso‐anal length were monitored at the end of the experiment. From the circumferences, naso‐anal length, and weight, the Lee Index, rate of body mass gain, and abdomen‐thorax ratio were determined as described by Novelli et al. (2007).

The animals were weighed every day at the same time. The animals were placed individually in a plastic container and then placed on a digital scale (Marconi, model BL3200H), deducting the weight of the container. Thoracic and abdominal circumference and naso‐anal length were measured using a tape measure. After anesthesia, the animal was held on a surface and its chest circumference was measured in the position behind the forelegs and its abdominal circumference in the position before the hind legs. To measure the naso‐anal length, marks were made on the surface coinciding with the animal's snout and the base of its tail, and then the distance in centimeters between the points was measured with a tape.

### 2.6 Glucose Tolerance Test

To check for metabolic alterations, the animals were subjected to the Glucose Tolerance Test (GTT). All the procedures carried out were based on the Guidelines and Considerations for Metabolic Tolerance Tests in Mice (Benedé‐Ubieto et al. 2020).

For the GTT, the animals were kept in clean polypropylene boxes, without food, and were only given water, fasting for 6 h. After the morning fasting period, they were weighed and administered a 20% glucose solution (D‐glucose anhydrous dextrose, Synth brand) intraperitoneally at a dose of 1.5 g of glucose per kg of body mass.

Blood samples were collected from the tail vessels through a small incision at the tip. Blood glucose levels were measured before the glucose solution was administered, as well as at 15, 30, 60, 90, and 120 min after glucose consumption, using Accu‐Chek Active blood glucose test strips (model GU Accu‐Chek). The results were expressed as the area under the glucose curve and the rate of serum glucose disappearance, respectively.

### 2.7 Fecal Moisture and pH

The analysis of feces moisture and pH (*n* = 5/group) was carried out according to the methodology of Batista et al. (2018). At the end of the experiment, fecal samples were collected and stored at −80°C for three consecutive days (Days 42, 43 and 44).

For moisture analysis, fecal samples were weighed in previously weighed crucibles and dried in an oven (SOLAB, model SL‐102, São Paulo, Brazil) with air circulation at 105°C overnight until constant weight. The result was obtained by subtracting the wet weight minus the dry weight. For fecal pH, the samples were diluted in distilled water (1 mg mL‐1) and the pH was measured using a bench pH meter (Hanna, model HI 99161, São Paulo, Brazil).

### 2.8 Microbiota Composition

To assess the composition of the intestinal microbiota, stool samples were collected inside the sanitized polypropylene cage using sterile tweezers, and the stool was stored in sterile eppendorfs at −80°C. DNA was then extracted using a commercial kit (QIAamp Fast DNA stool mini kit, Qiagen), according to the manufacturer's protocol.

The quality of the DNAs was checked using the FastQC program (Andrews 2010). Additionally, the libraries were processed using the functions *fastx_info* and *fastq_eestats2* from the USEARCH software (v.11.0.667; Edgar 2010) to assess quality score distributions, sequence length profiles, and expected error rates. The *search_oligodb* function from the same software was employed using the primer pair targeting the V3–V4 region of the 16S rRNA gene (341F: 5′‐CCTACGGGNGGCWGCAG‐3′; 805R: 5′‐GACTACHVGGGTATCTAATCC‐3′) to identify their presence and positions within the reads.

Based on this analysis, heterogeneous bases were detected upstream of the primer sequences. Therefore, both the heterogeneous regions and the amplification primers were removed using Atropos (v.1.1.31; Didion et al. 2017). The 3′ ends of the sequences were further trimmed to improve overall read quality. For this purpose, Fastp (v.0.23.2; Chen et al. 2018) was used to remove bases when a sliding window of five consecutive nucleotides (*−cut_right_window_size 5*) presented a mean Phred quality score below 25 (*−cut_right_mean_quality 25*).

In addition, reads with an average quality score below Q25 (*−average_qual 25*) and those shorter than 230 bp (*−length_required 230*) were discarded. Finally, paired‐end reads (R1 and R2) were merged by overlap using FLASH (v.1.2.11; Magoč and Salzberg 2011), with a minimum overlap of 10 base pairs (*−min‐overlap 10*).

Merged reads were processed using the DADA2 pipeline (Callahan et al. 2016) implemented in the *dada2* R package (v.1.22.0) within the R statistical environment R Core Team 2021. Initially, the readings were filtered and truncated by the *filterAndTrim* function, considering an expected error of 3 (*maxEE = 3*). Next, the probability of errors in the bases was estimated (*learnErrors*) and the sequences were corrected based on the model obtained (*dada*). In this way, Amplicon Sequence Variants (ASVs) present in each sample were designated, investigated and filtered for possible chimeric sequences (*removeBimeraDenovo*). The ASVs were annotated in the NCBI RefSeq 16S sequence reference database supplemented with PDR to classify them taxonomically (Cole et al. 2005). ASVs not classified as Bacteria or Archaea, as well as those identified as host‐associated contaminants (e.g., mitochondrial sequences), were removed. Additionally, ASVs present in only a single replicate were excluded from downstream analyses.

The phylogenetic relationship of the ASVs was established using the Neighbor‐Joining algorithm, and then statistically evaluated using the bootstrap method from the R package *phangorn*. The counts, taxonomic annotations, and the phylogenetic tree of the ASVs were exported in *phyloseq* format. The phyloseq object was then transformed into compositional data from the R package *metagMisc* to carry out the microbiome analyses (McMurdie and Holmes 2013; Mikryukov 2022; Schliep 2011).

### 2.9 Blood Biochemical Analysis

Liver tissue was isolated following the sacrifice of the mice (*n* = 5/group), fixed for 24 h in a 4% formaldehyde solution, processed using a histological processor (MTP 100 Slee, Mainz, Germany), embedded in paraffin wax, and sectioned at 4 μm with a Hyrax M60 microtome (Carl Zeiss, Germany).

The tissue sections were placed on glass slides for histological staining. For histopathological analysis, the tissue was stained with hematoxylin and eosin (H&E). For glycogen analysis, the tissue was stained with periodic acid‐Schiff (PAS) at 1%.

### 2.10 Statistical Analysis

The results of the sample characterization are expressed as mean ± standard deviation. The results obtained from the in vivo experimental analysis are expressed as mean values ± standard error of the mean. The results obtained in all stages were submitted to Analysis of Variance (ANOVA) and Bonferroni (*p* < 0.05), and Tukey's test will be used to compare the means (*p* < 0.05) within the experimental groups.

To analyze the intestinal microbiota, the effectiveness of sampling was assessed using rarefaction curves, based on the “amp_rarecurve” analysis from the R package “ampvis2” (Andersen et al. 2018). Alpha‐diversity was estimated by examining observed richness and diversity metrics (Shannon index, Gini–Simpson index, and Faith's Phylogenetic Diversity (PD)). The measurements obtained were statistically compared using the Kruskal‐Wallis test (*p*‐value < 0.1). For the indices that showed a significant difference between the groups, the Fisher's LSD post hoc test was used for the pairwise comparison and grouping of the means (*p*‐value < 0.1).

## 3 Results and Discussion

### 3.1 Chemical Characterization of Araticum By‐Product

The proximate composition and technological characterization of the by‐product are presented in Table 1. The araticum by‐product stands out for its high total fiber content—nearly 50% of the by‐product composition, mostly consisting of insoluble fibers, when compared to araticum pulp (3.56 g 100 g^−1^) (Dragano et al. 2010).

**TABLE 1:** Proximate composition (g 100 g^−1^) and technological characterization of the araticum by‐product.

Proximate composition (g 100 g^−1^)	Mean ± DP
Moisture	8.07 ± 0.06
Protein	11.17 ± 0.69
Lipids	9.79 ± 0.22
Total fibers	53.29 ± 0.54
Soluble fibers	3.92 ± 0.04
Insoluble fibers	49.37 ± 0.53
Ashes	2.73 ± 0.02
Carbohydrates^a^	14.95 ± 0.20
Technological characterization	
Water absorption index (g g^−1^ de flour)	4.22 ± 0.13
Water solubility index (%)	15.76 ± 0.21
Oil absorption index (g gel g^−1^ de flour)	7.82 ± 2.68

*Note:* Mean ± standard deviation (*n* = 4).

^a^ Calculated as 100—(moisture + ash + protein + lipids + total dietary fiber).

The protein and lipid content is also higher in the peel compared to the pulp (average protein: 1.39 g 100 g^−1^; average lipids: 3.8 g 100 g^−1^). As expected, the pulp has a higher moisture content than the by‐product, with an average of 72.43 g 100 g^−1^ (Dragano et al. 2010; Roesler et al. 2007; Silva et al. 2008).

The centesimal composition results of the araticum by‐product highlighted that consuming 100 g of the araticum by‐product provides approximately 53% total fiber, which would meet the daily recommended intake of dietary fiber for healthy adults, approximately 25 to 30 g (Brazil 2022).

Dietary fiber provides a wide range of health benefits, including promoting satiety, improving glycemic control, lowering serum cholesterol levels, and supporting intestinal health. Beyond these well‐established physiological effects, fibers also play a crucial role in maintaining gut homeostasis by serving as substrates for microbial fermentation. The extent and nature of these effects are strongly influenced by the physicochemical characteristics of the fiber, such as solubility, viscosity, and fermentability, which determine its interaction with the gut environment and resident microbiota (Arruda and Pastore 2019; Holscher 2017; Slavin 2013). Fermentable fibers, in particular, stimulate the growth of beneficial bacteria and the production of short‐chain fatty acids (SCFAs), metabolites that contribute to improved intestinal barrier function, immune modulation, and metabolic regulation (Koh et al. 2016; Makki et al. 2018). Therefore, identifying novel fiber sources with favorable functional and fermentative properties is essential for developing strategies to improve metabolic health and prevent diet‐related disorders.

Regarding the technological properties of the analyzed by‐product, the water absorption index was found to be relatively low, whereas the oil absorption index showed more favorable values. The low water absorption suggests that this flour may not be suitable for formulations requiring high hydration, such as breads or cakes; however, it could be advantageous in products like cookies and dry doughs, where limited water retention is desirable. Additionally, the low water solubility may contribute positively to product stabilization and textural properties in certain food applications (Föste et al. 2020).

### 3.2 Blood Biochemical Analysis

Animals fed a high‐fat diet exhibited a markedly higher weight gain (162%) compared with the control group, confirming the obesogenic effect of the diet. Supplementation with the araticum by‐product at both tested concentrations (HFD‐A200 and HFD‐A400) did not result in significant differences in weight gain compared with either the HFD or control groups. These findings suggest that, under the experimental conditions, the inclusion of the araticum by‐product did not exert a hypocaloric or anti‐obesogenic effect sufficient to counteract the impact of the high‐fat diet.

Despite its high fiber content, particularly insoluble fiber, which is typically associated with increased satiety and reduced energy intake (Slavin 2013; Reynolds et al. 2020), the amount and duration of supplementation may not have been sufficient to elicit measurable changes in body mass. It is also possible that compensatory metabolic adaptations or limited fermentability of the fiber fraction influenced these outcomes (Makki et al. 2018).

The murinometric parameters of the experimental groups are summarized in Table 2. Thoracic and abdominal circumferences, as well as naso‐anal length, showed no significant differences among groups, indicating that the 30‐day dietary intervention did not markedly alter body composition. Together, these results demonstrate that short‐term supplementation with the araticum by‐product did not modify growth or morphometric parameters, although other metabolic and intestinal effects may still occur independent of changes in body weight (Koh et al. 2016).

**TABLE 2:** Effect of high‐fat diet and Araticum by‐product supplementation on murinomeric parameters of experimental groups.

Parameters	GROUPS			
	CTRL	HFD	HFD‐A200	HFD‐A400
Weight gain^a^	6.77 ± 2.08B	17.77 ± 3.54A	13.69 ± 2.95AB	11.24 ± 2.71AB
TC^b^	8.53 ± 0.19A	8.90 ± 0.23A	8.70 ± 0.18A	8.75 ± 0.18A
AC^c^	9.65 ± 0,24A	10.17 ± 0.26A	9.98 ± 0.19A	9.83 ± 0.15A
NL^d^	10.27 ± 0.08A	10.45 ± 0.10A	10.23 ± 0.08A	10.18 ± 0.06A
Lee index	23.53 ± 0.80B	29.15 ± 1.60A	25.77 ± 1.25AB	25.17 ± 0.72AB
Glycemia	122 ± 8.27B	158 ± 9.69A	149 ± 5.11A	153 ± 4.03A

*Note:* Mean ± standard deviation (*n* = 7). Values are expressed as mean ± standard error of the mean. Different uppercase letters in the same row indicate statistically significant differences (*p* < 0.05). CTRL: Control. HFD: High‐fat diet. HFD‐A200: High‐fat diet treated with 200 mg kg^−1^ of araticum by‐product. HFD‐A400: High‐fat diet treated with 400 mg kg^−1^ of araticum by‐product.

^a^ Expressed in g 100 g^−1^.

^b^ Thoracic circumference—expressed in cm.

^c^ Abdominal circumference, expressed in cm.

^d^ Nose‐to‐anus length, expressed in cm.

**FIGURE 1:**
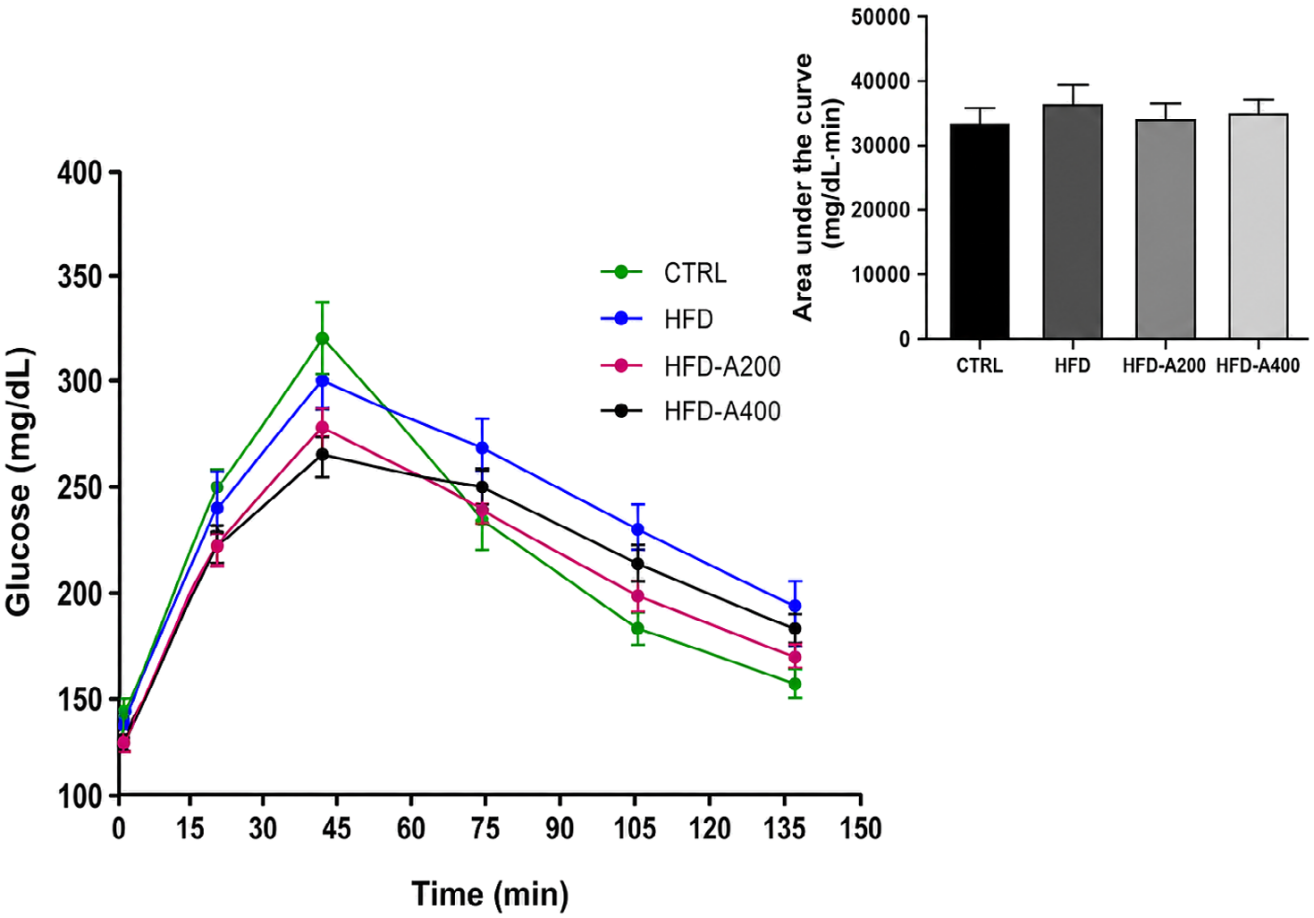
Glucose tolerance test in mice from the CTRL, HFD, HFD‐A200, and HFD‐A400 groups. Values are expressed as mean ± standard error of the mean. No significant differences were observed among the groups by ANOVA (*p* > 0.05). CTRL: Control diet; HFD: High‐fat diet; HFD‐A200: High‐fat diet supplemented with 200 mg kg^−1^ of araticum by‐product; HFD‐A400: High‐fat diet supplemented with 400 mg kg^−1^ of araticum by‐product.

Regarding the glucose tolerance test, there was no significant difference among the groups (Figure 1), indicating that the araticum by‐product had no direct impact on glucose tolerance at the evaluated doses.

**FIGURE 2:**
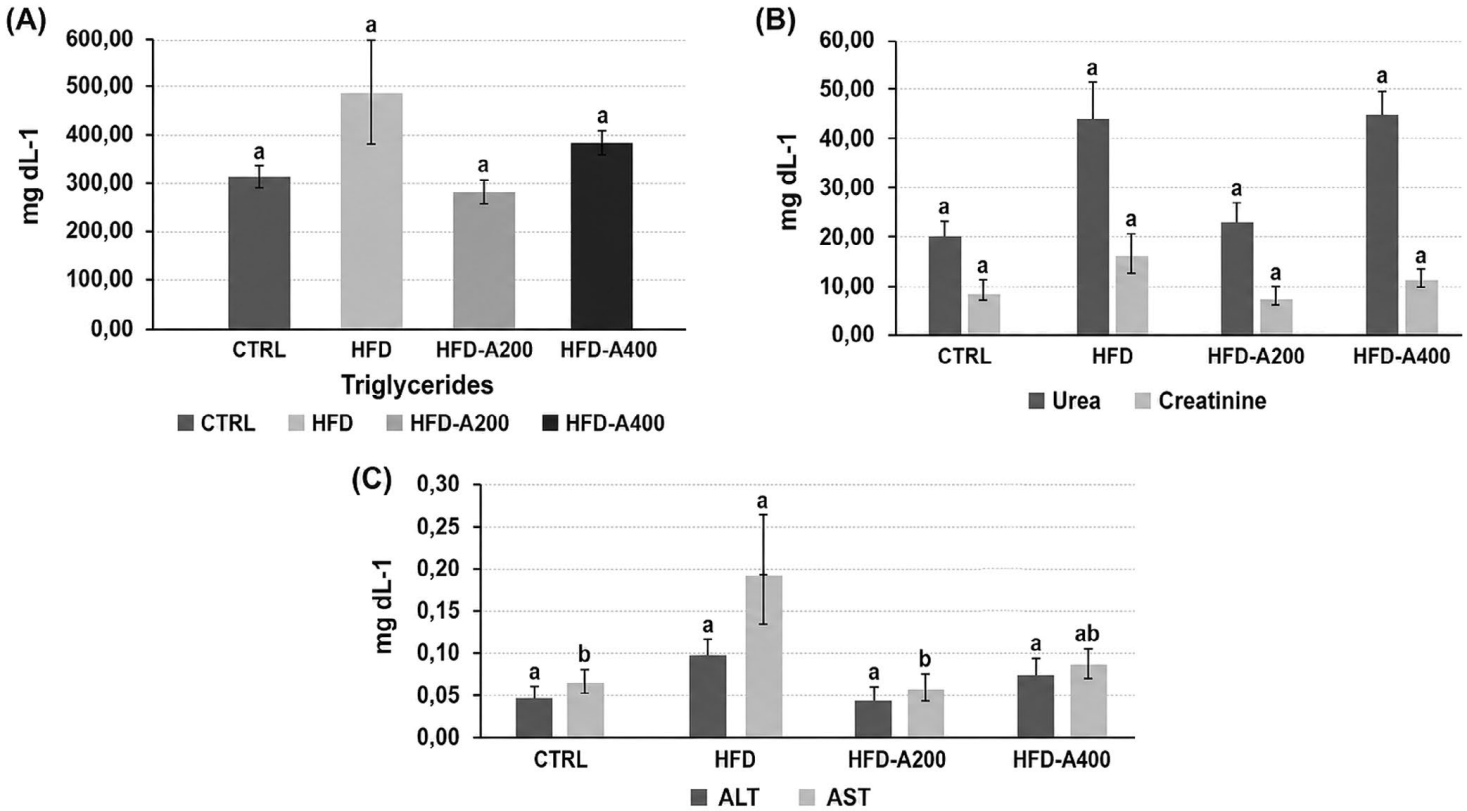
Triglycerides (A), Urea and Creatinine (B), Alanine aminotransferase (ALT), Aspartate aminotransferase (AST) (C) of control animals (CTRL), animals on a high‐fat diet (HFD), and animals given 200 mg Kg^−1^ (HFD‐A200) and 400 mg Kg^−1^ (HFD‐A400) of araticum by‐product. Values are expressed as mean ± standard error of the mean represented by vertical bars. Different letters in the same line show statistical differences by ANOVA and Bonferroni (*p* < 0.05).

Among the biochemical tests, the HFD group showed elevated AST levels compared to the CTRL and HFD‐A200 groups, suggesting a possible hepatic alteration in these animals fed a high‐fat diet (Figure 2). However, AST is not a liver‐specific enzyme and may also reflect extrahepatic sources; therefore, this finding should be interpreted with caution. Notably, the group with the highest dose of by‐product (HFD‐A400) did not differ from the HFD group, indicating that the higher dose of the by‐product did not exacerbate AST levels beyond those observed with the high‐fat diet alone. Nevertheless, these findings raise potential concerns regarding liver safety at higher doses of the araticum by‐product. The presence of bioactive compounds such as alkaloids (stephalanine, liriodenine, atherospermidine, isopiline) previously identified in the peel suggests that dose‐dependent effects cannot be ruled out. Although their direct impact on hepatic function was not evaluated in this study, these compounds have been associated with biological activity and may contribute to hepatic responses.

Overall, while no definitive evidence of hepatotoxicity was observed, the results highlight the need for careful dose evaluation and further safety assessment. Future studies including a broader panel of liver function markers and detailed histopathological analyses are necessary to better characterize potential hepatic effects and establish safe intake levels (Arruda et al. 2023).

### 3.3 Histological Analysis

**FIGURE 3:**
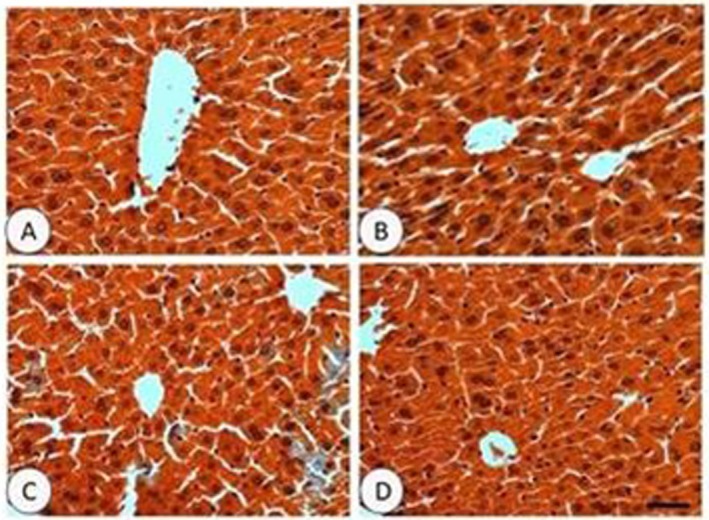
Effect of high‐fat diet and consumption of Araticum by‐product on liver morphology. (A) CTRL group. (B) HFD group. (C) HFD‐A200 group. (D) HFD‐A400 group. No differences were observed among the groups. Staining: HE, Bar: 10 μm.

**FIGURE 4:**
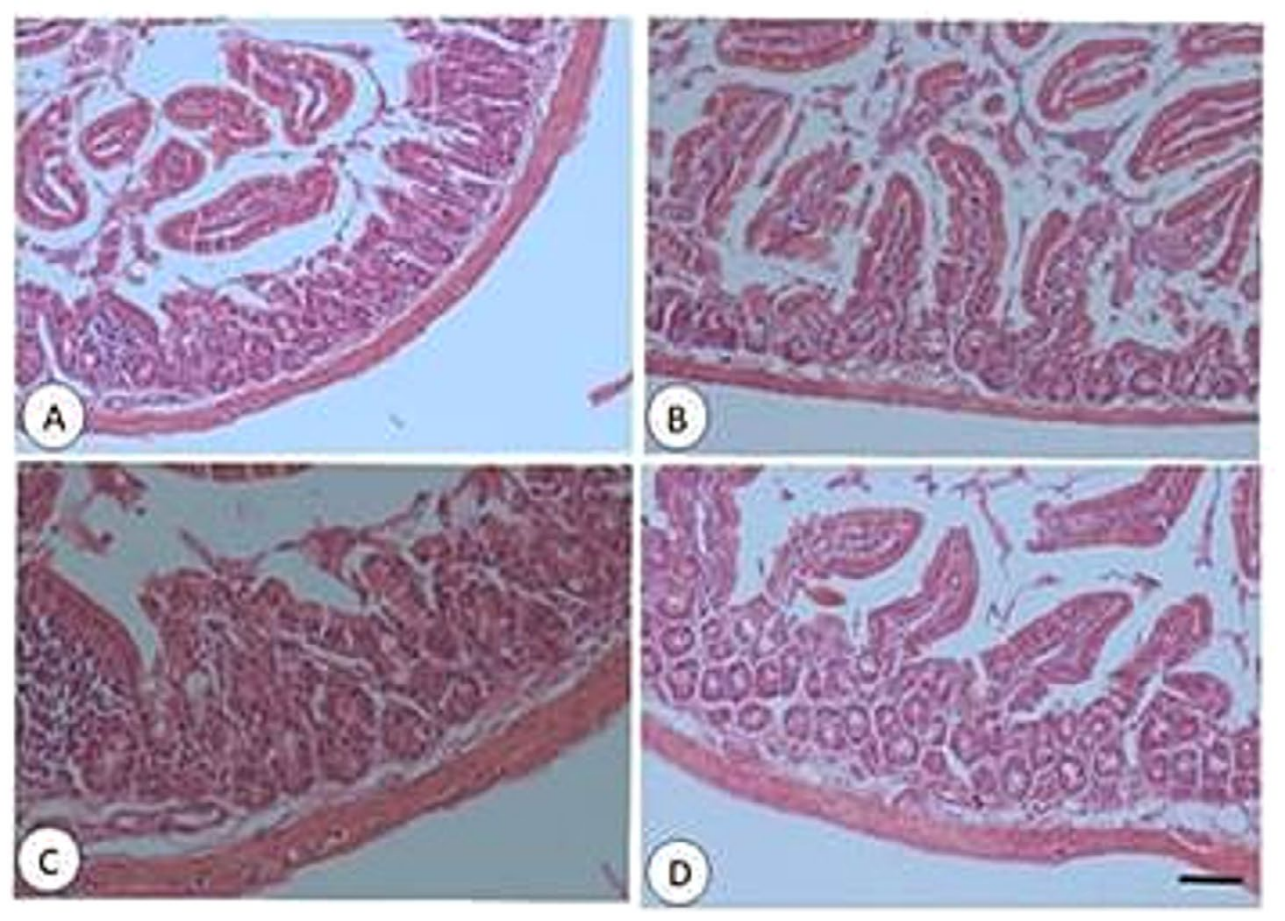
Effect of high‐fat diet and consumption of araticum by‐product on intestinal morphology. (A) CTRL group. (B) HFD group. (C) HFD‐A200 group. (D) HFD‐A400 group. No differences were observed among the groups. Staining: HE, bar: 10 μm.

Histological analyses of the liver and intestine showed no significant alterations among the experimental groups (Figures 3 and 4). Despite variations in the treatments, no evident structural damage was observed. Furthermore, it was noted that the morphological characteristics of the intestinal villi and crypts were maintained, supporting the idea that the treatments did not induce significant changes. The preservation of these structures is essential for maintaining proper intestinal function, including nutrient absorption and protection against pathogens.

**FIGURE 5:**
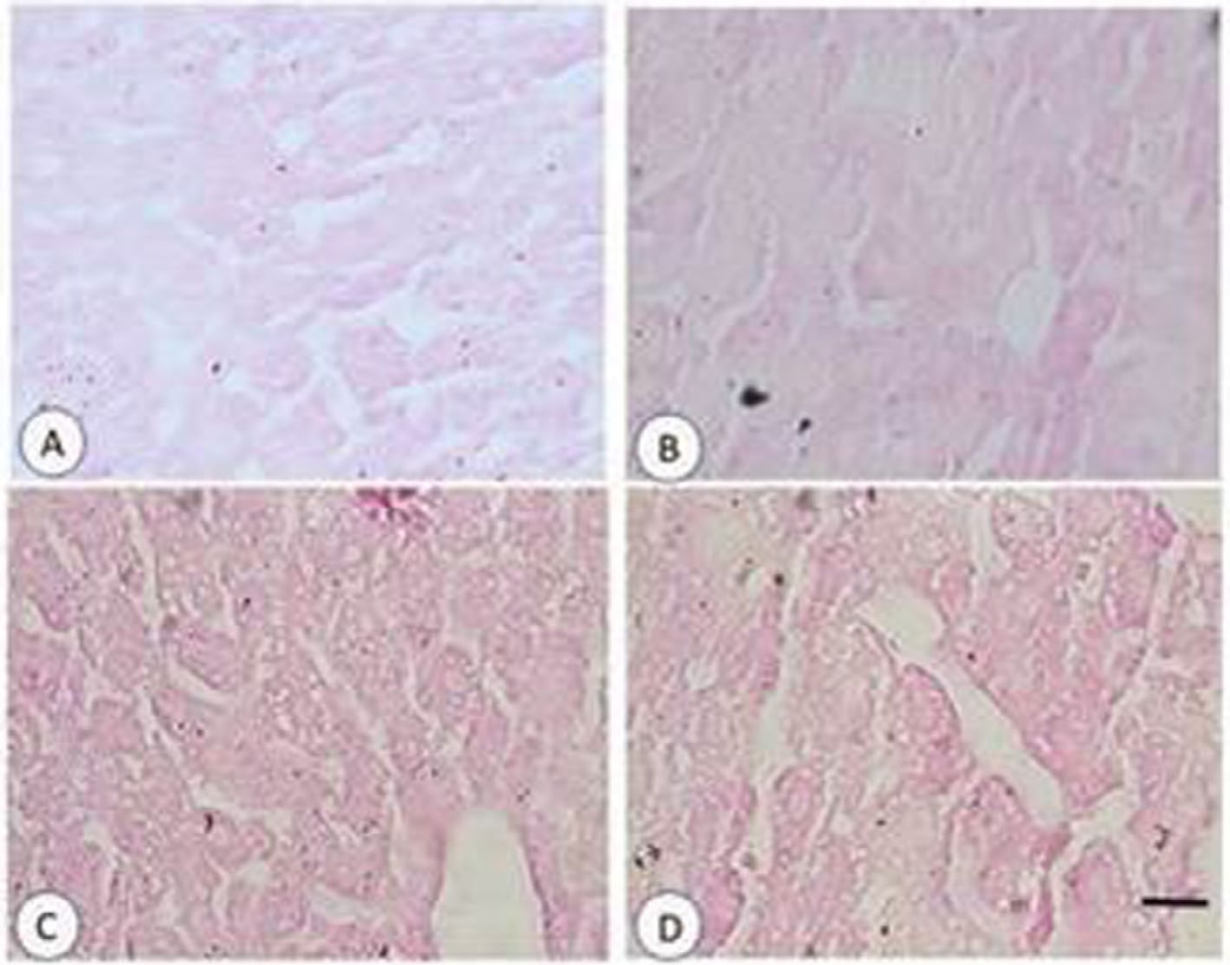
Effect of high‐fat diet and consumption of araticum by‐product on liver glycogen identification. (A) CTRL group showing basal staining. (B) HFD group and (C) HFD‐A200 group showed moderate staining. (D) HFD‐A400 group showed high staining. Staining: PAS. Bar: 5 μm.

**FIGURE 6:**
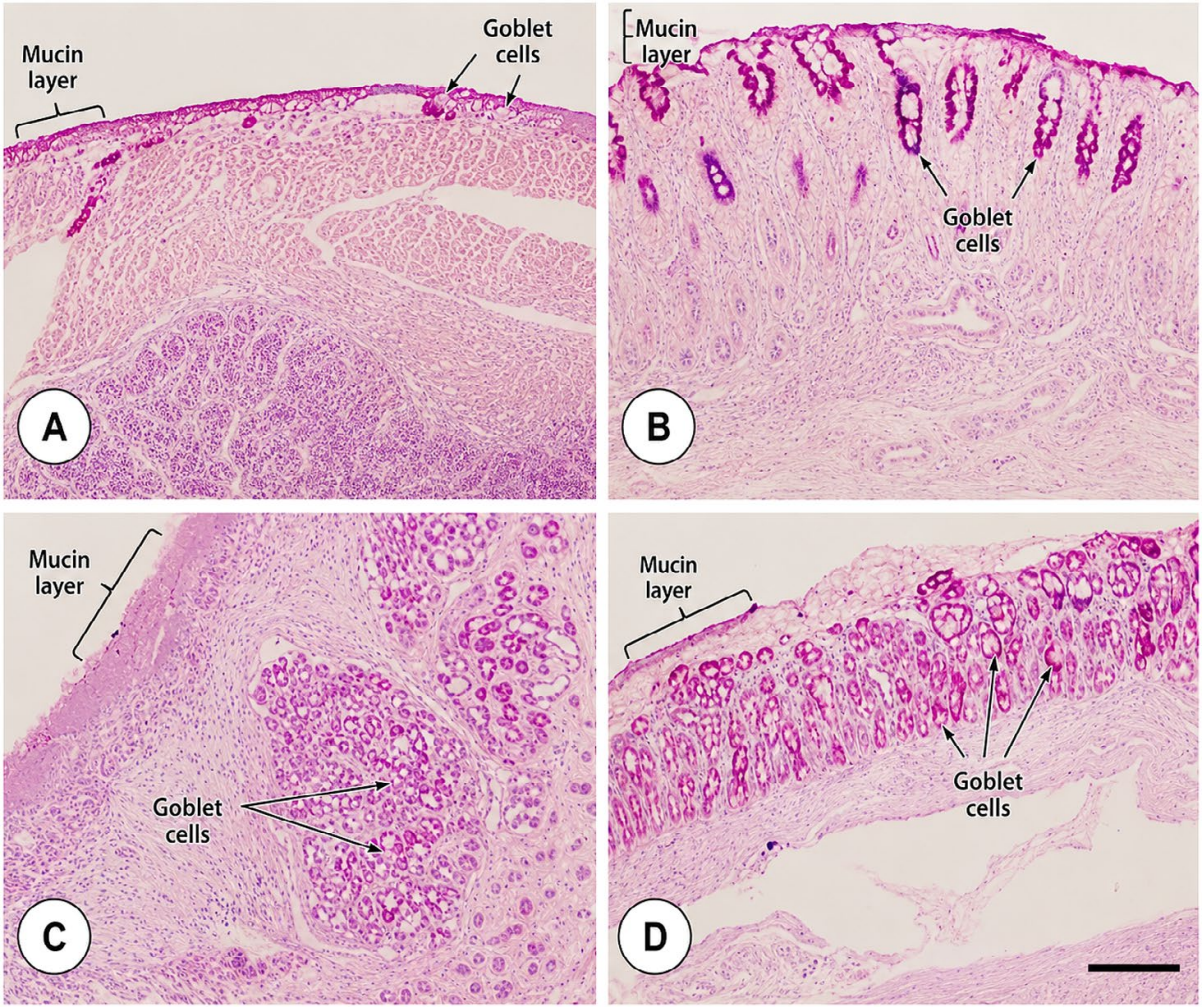
Effect of the high‐fat diet and Araticum by‐product supplementation on PAS‐positive structures in intestinal tissue. (A) CTRL group, (B) HFD group, (C) HFD‐A200 group, and (D) HFD‐A400 group. Arrows indicate PAS‐positive goblet cells and mucin‐containing regions. More intense PAS staining was observed in the HFD‐A400 group. PAS staining. Scale bar: 20 μm.

The PAS (periodic acid‐Schiff) staining in the liver and intestine is shown in Figures 5 and 6. In both tissues, a basal glycogen staining was identified in the CTRL group, a moderate staining in the HFD and HFD‐A200 groups, and intense staining in the HFD‐A400 group.

In the hepatic tissue, this result demonstrates an increased glycogen deposition, caused by the high‐fat diet and/or the by‐product. This is corroborated by the biochemical AST analysis, suggesting hepatic damage or alterations in glycogen metabolism.

Regarding the intense staining observed in the intestinal tissue of the HFD‐A400 group, this finding may reflect an increased presence of mucins, highly glycosylated proteins secreted by goblet cells. Mucins are fundamental components of the mucus layer that covers the intestinal epithelium, playing a key role in preserving barrier integrity, protecting epithelial cells against mechanical and chemical damage, and modulating host–microbe interactions. An abundant mucus layer contributes to intestinal homeostasis by preventing direct contact between luminal bacteria and epithelial cells while simultaneously providing binding sites and nutrients that support the colonization of commensal microorganisms (Paone and Cani 2020).

The more intense PAS staining observed in the HFD‐A400 group may indicate a greater presence of PAS‐positive glycoproteins, including mucins. However, because mucin content and goblet cell numbers were not quantitatively assessed, this finding cannot be interpreted as direct evidence of increased mucin production. The enhanced mucin production observed in the HFD‐A400 group may suggest a protective adaptive response, possibly associated with the high fiber content of the araticum by‐product. Dietary fiber fermentation and the resulting production of metabolites such as butyrate represent possible mechanisms that could influence goblet cell differentiation and mucin synthesis (Gaudier et al. 2004; Willemsen et al. 2003). However, mucin levels and SCFA concentrations were not directly measured in this study, and therefore these mechanisms remain hypothetical.

Thus, while the araticum by‐product may contribute to maintaining intestinal barrier function and epithelial protection under a high‐fat diet, these interpretations should be made with caution and require confirmation through targeted functional analyses. Future studies should directly quantify intestinal mucins and fecal and/or cecal SCFAs to confirm whether these mechanisms are involved in the observed histological changes.

### 3.4 Fecal Water Retention and Intestinal pH

The results regarding fecal characteristics are presented in Table 3. Supplementation with araticum by‐product flour promoted a significant increase in fecal moisture, particularly in the HFD‐A400 group, which exhibited the highest value (49.12% ± 1.98%) compared with the control and HFD groups. This effect is attributed to the high content of insoluble fibers in the araticum by‐product, which increases water retention capacity and fecal bulk, thereby stimulating intestinal peristalsis and accelerating transit time (Batista et al. 2018; Bijkerk et al. 2004; Hu et al. 2012). Similar findings were reported in dyslipidemic rats treated with acerola, cashew, and guava by‐products, where acerola fiber promoted greater fecal moisture, highlighting the role of fruit‐derived fibers in improving stool hydration (Batista et al. 2018).

**TABLE 3:** Effect of high‐fat diet and Araticum by‐product supplementation on fecal parameters (moisture and pH) in mice.

	Groups			
	CTRL	HFD	HFD‐A200	HFD‐A400
Moisture	37.89 ± 1.22B	39.72 ± 2.81B	42.75 ± 1.85AB	49.12 ± 1.98A
pH	8.13 ± 0.03A	8.04 ± 0.12AB	7.52 ± 0.03B	7.61 ± 0.23AB

*Note:* Mean ± standard deviation (*n* = 5). Different letters in the same line show statistical differences by ANOVA and Bonferroni (p < 0.05).

Insoluble fibers act mainly by absorbing water and increasing fecal mass, while soluble fibers tend to form viscous gels that delay gastric emptying and glucose absorption (Slavin 2013; Holscher 2017). The predominance of insoluble fiber in the araticum by‐product (approximately 49 g 100 g^−1^) likely explains the observed increase in fecal water content without compromising intestinal consistency. From a nutritional standpoint, such properties are advantageous, as they contribute to regular bowel movements and may help prevent constipation effects commonly associated with high‐fiber diets.

With respect to fecal pH, the control group exhibited a higher mean value (8.13 ± 0.03), differing significantly only from the HFD‐A200 group (*p* < 0.05). Both araticum‐treated groups showed a tendency toward reduced fecal pH, which may be related to the fermentative activity of indigestible carbohydrates present in the by‐product. The microbial fermentation of these substrates can lead to the production of short‐chain fatty acids (SCFAs), such as acetate, propionate, and butyrate, which lower luminal pH and contribute to intestinal health (Holscher 2017). However, SCFA concentrations were not directly measured in this study; therefore, this interpretation should be considered hypothetical.

Although the araticum by‐product contains relatively low amounts of soluble fiber, typically more fermentable than insoluble type, the observed tendency toward a lower fecal pH is compatible with, but does not demonstrate, microbial fermentation of components present in the by‐product. In addition, SCFAs have been associated with epithelial integrity, immune modulation, and energy supply to colonocytes (Makki et al. 2018); however, these mechanisms cannot be confirmed in the present study and therefore this interpretation should be considered hypothetical. Therefore, direct quantification of fecal and/or cecal SCFAs is required in future studies to confirm whether microbial fermentation contributed to the reduction in fecal pH. Thus, while the inclusion of araticum by‐product was associated with changes in fecal pH and moisture, the physiological significance of these changes requires confirmation through direct metabolic and functional measurements.

### 3.5 Microbiota Composition

**FIGURE 7:**
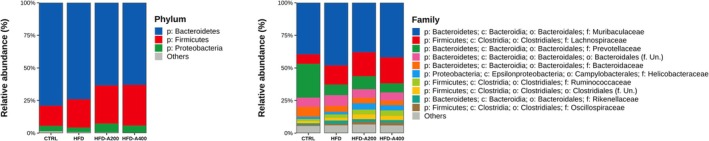
Taxonomic profiles of the gut microbial communities in the different experimental groups. Relative abundance at the phylum level (A) and family level (B). Taxa with a mean relative abundance below [1%] were grouped as “Others.” CTRL: Control diet; HFD: High‐fat diet; HFD‐A200: High‐fat diet supplemented with 200 mg kg^−1^ of araticum by‐product; HFD‐A400: High‐fat diet supplemented with 400 mg kg^−1^ of araticum by‐product.

The taxonomic profile of the bacteria community summarizes the most representative taxa identified under each treatment condition. In the microbiota of mice, the predominant phyla were Bacteroidetes, Firmicutes, and Proteobacteria across all groups, including the control (CTRL) and those supplemented with the araticum by‐product (HFD‐A200 and HFD‐A400) (Figure 7).

A trend toward a lower relative proportion of Bacteroidetes and a higher proportion of Firmicutes was noted in the groups supplemented with the araticum by‐product (HFD‐A200 and HFD‐A400) compared with the control group (Figure 7). These shifts may reflect changes in substrate availability, particularly related to the presence of dietary fibers, which can influence microbial community structure (Sun et al. 2023; Holscher 2017).

Some members of the Firmicutes phylum possess a remarkable ability to utilize complex polysaccharides as energy sources. This process begins with the binding of dietary fibers through carbohydrate‐ or solute‐binding proteins located on the bacterial cell surface, allowing monosaccharides and oligosaccharides to be transported into the cytoplasm. Once internalized, these compounds are metabolized into simpler molecules that fuel bacterial energy metabolism (Sun et al. 2023).

However, the interpretation of changes at the phylum level should be approached with caution. The Firmicutes phylum encompasses a highly diverse group of taxa with distinct and sometimes contrasting metabolic functions, and therefore an increase in its relative abundance cannot be directly interpreted as beneficial. While some Firmicutes members are involved in the fermentation of complex polysaccharides and the production of metabolites relevant to intestinal physiology, these functional attributes are not uniform across the phylum (Magne et al. 2020; Vacca et al. 2020). Thus, the observed compositional shifts should be interpreted as indicative of microbial restructuring in response to dietary supplementation, rather than as evidence of specific functional or health‐related outcomes.

Within the Bacteroidetes phylum, the *Prevotellaceae* family was relatively more represented in the CTRL group (22.44%) compared with the other groups (HFD: 8.12%; HFD‐A200: 10.0%; HFD‐A400: 6.99%). These differences suggest that the high‐fat diet may have altered the taxonomic composition of the gut microbiota, shifting its composition independently of the araticum by‐product supplementation. Previous studies have associated carbohydrate‐ and fiber‐rich diets with higher *Prevotellaceae* abundance, often linked to enhanced fermentation capacity and metabolic flexibility of the gut microbiota (Jiang et al. 2022). The distinct microbial patterns observed here align with evidence that dietary macronutrient composition, particularly fat content, can strongly influence microbial ecology, sometimes overriding the effects of specific fiber interventions.

Among bacterial families, *Muribaculaceae* showed the highest overall relative abundance, with an average composition of 41.42% across treatments (Figure 7). This family has previously been associated with intestinal characteristics such as epithelial barrier integrity, goblet cell activity, mucin secretion, and mucus glycosylation. However, these functions were not evaluated in the present study, and the relative abundance of Muribaculaceae alone does not demonstrate functional or health‐related effects (Paone and Cani 2020). Interestingly, previous studies demonstrated that supplementation with fructooligosaccharides (FOS) in C57BL/6J mice not only mitigated the adverse metabolic effects of a high‐fat diet but also enhanced the relative abundance of *Muribaculaceae*, reinforcing its potential role in maintaining gut health under obesogenic conditions (Paone et al. 2022). Nevertheless, functional microbiome analyses would be required to determine whether similar metabolic activities occurred in the present experimental model.

Richness and alpha diversity, using Shannon index, Gini‐Simpson index, and Faith's phylogenetic diversity, were evaluated (Table 4). Richness, which represents the total number of distinct taxa detected per sample, tended to be higher in the groups receiving araticum by‐product supplementation (HFD‐A200 and HFD‐A400) compared with the control. However, this difference did not reach statistical significance (*p* = 0.07). No statistical differences were observed in the Shannon, Gini‐Simpson, or Faith's PD indices. Although a numerical tendency toward higher richness was observed in the supplemented groups, this difference was not statistically significant and should not be interpreted as evidence that the intervention increased microbial diversity (Winer et al. 2016).

**TABLE 4:** Effect of high‐fat diet and Araticum by‐product supplementation on gut microbiota richness and alpha‐diversity (Shannon, Gini–Simpson and Faith's phylogenetic diversity) in mice.

Group	Richness	Shannon	Gini‐Simpson	Faith's PD
CTRL	133.57 ± 22.21B	3.81 ± 0.21A	0.95 ± 0.02A	23.37 ± 3.02A
HFD	144.00 ± 22.70AB	4.06 ± 0.20A	0.97 ± 0.01A	26.40 ± 4.74A
HFD‐A200	165.57 ± 24.39A	4.15 ± 0.38A	0.96 ± 0.02A	27.13 ± 3.06A
HFD‐A400	157.71 ± 29.56A	4.12 ± 0.35A	0.97 ± 0.02A	27.65 ± 5.55A

*Note:* Mean ± standard deviation (*n* = 7). Different uppercase letter columns represent statistical differences between the means observed in the treatments.

Although the tested doses were effective in promoting intestinal changes in the experimental model, direct translation to human intake is not straightforward. Dose extrapolation from animal studies must consider differences in body size, metabolism, intestinal physiology, microbiota composition, and dietary background. Thus, the present findings should be interpreted as preclinical evidence supporting future studies designed to establish human‐equivalent intake ranges, long‐term safety, and clinical relevance.

Despite the relevant findings reported herein, some limitations should be acknowledged when interpreting the results. First, the duration of the intervention may have been insufficient to elicit more pronounced changes in body weight, glucose homeostasis, and other systemic metabolic parameters. Second, as this study was conducted in an experimental animal model, direct extrapolation of the findings to human nutrition and health should be approached with caution. Third, although changes in intestinal parameters and gut microbiota composition were observed, short‐chain fatty acids and other fermentation‐derived metabolites were not directly quantified, which limits the mechanistic interpretation of the findings. Future studies should directly quantify fecal and/or cecal SCFAs to confirm the proposed association between fiber fermentation, fecal pH, and intestinal responses. Furthermore, microbiota‐related findings, particularly those at the phylum level, should be interpreted carefully, as compositional shifts do not necessarily correspond to functional alterations. Finally, the liver‐related biochemical results require further investigation, ideally including additional biomarkers and histopathological analyses. Taken together, these limitations indicate that the present findings should be regarded as preliminary evidence of intestinal modulation under the conditions evaluated, rather than as definitive proof of anti‐obesity efficacy or direct clinical relevance. Confirmation in longer‐term animal studies and subsequently in well‐controlled human studies is necessary before conclusions regarding clinical relevance or health‐promoting effects can be drawn.

## 4 Conclusion

In conclusion, the araticum by‐product is a source of predominantly insoluble dietary fiber and bioactive compounds. Under the conditions evaluated, supplementation did not significantly affect body weight, glucose tolerance, or most biochemical and murinometric parameters in mice fed a high‐fat diet. The intervention was associated with increased fecal moisture, a tendency toward lower fecal pH, more intense intestinal PAS staining at the highest dose, and changes in the relative abundance of selected microbial taxa. However, SCFA production, mucin content, intestinal barrier function, and microbial metabolic activity were not directly assessed. Therefore, the findings should be interpreted as preliminary preclinical evidence of changes in selected intestinal parameters rather than proof of anti‐obesity, metabolic, or microbiota‐mediated health benefits. Longer‐term studies incorporating direct SCFA quantification, quantitative mucin assessment, intestinal permeability markers, and functional microbiome analyses are required before the applicability of the araticum by‐product as a functional food ingredient can be established.

## Author Contributions

**Sabrina Neves Casarotti:** conceptualization, investigation, funding acquisition, methodology, writing – review and editing, project administration, data curation, supervision, resources. **Eudes Thiago Pereira Avila:** methodology, writing – review and editing, formal analysis. **Fellipe Lopes de Oliveira:** investigation, methodology, formal analysis, data curation. **Daniel Guariz Pinheiro:** formal analysis, software, methodology, data curation, writing – review and editing. **Lucas Amoroso Lopes de Carvalho:** writing – review and editing, formal analysis, software, data curation. **Katia Sivieri:** formal analysis, data curation, writing – review and editing. **Thamara Rosa de Souza:** investigation, writing – original draft, methodology, writing – review and editing, formal analysis, software, validation. **Suélem Aparecida de França Lemes:** funding acquisition, methodology, formal analysis, project administration, supervision. **Amílcar Sabino Damazo:** writing – review and editing, methodology, formal analysis. **Maressa Caldeira Morzelle:** conceptualization, investigation, funding acquisition, writing – original draft, methodology, validation, writing – review and editing, data curation, supervision, resources, project administration.

## Funding

This research was supported by the Conselho Nacional de Desenvolvimento Científico e Tecnológico – CNPq, Brazil: 426153/2018‐9.

## Conflicts of Interest

The authors declare no conflicts of interest.

## Data Availability

The data that support the findings of this study are available from the corresponding author upon reasonable request.

## References

[fsn372163-bib-0059] Andersen, K. S. , R. H. Kirkegaard , S. M. Karst , and M. Albertsen . 2018. “ampvis2: An R Package to Analyse and Visualise 16S rRNA Amplicon Data.” bioRxiv. 10.1101/299537.

[fsn372163-bib-0001] Anderson, R. A. , H. F. Conway , and A. J. Peplinski . 1970. “Gelatinization of Corn Grits by Roll Cooking, Extrusion Cooking and Steaming.” Starch/Staerke 22: 130–135. 10.1002/star.19700220408.

[fsn372163-bib-0002] Andrews, S. 2010. FastQC: A Quality Control Tool for High Throughput Sequence Data. Babraham Institute.

[fsn372163-bib-0003] Arruda, H. S. , F. T. Borsoi , A. C. Andrade , G. M. Pastore , and M. R. Maróstica Júnior . 2023. “Scientific Advances in the Last Decade on the Recovery, Characterization, and Functionality of Bioactive Compounds From the Araticum Fruit (*Annona crassiflora* Mart.).” Plants 12: 1536. 10.3390/plants12071536. 37050162 PMC10097317

[fsn372163-bib-0004] Arruda, H. S. , and G. M. Pastore . 2019. “Araticum (*Annona crassiflora* Mart.) as a Source of Nutrients and Bioactive Compounds for Food and Non‐Food Purposes: A Comprehensive Review.” Food Research International 123: 450–480. 10.1016/j.foodres.2019.05.011. 31284996

[fsn372163-bib-0005] Association of Official Analytical Chemists . 1995. Official Methods of Analysis of AOAC International. AOAC.

[fsn372163-bib-0006] Batista, K. S. , A. F. Alves , M. S. Lima , et al. 2018. “Beneficial Effects of Consumption of Acerola, Cashew or Guava Processing By‐Products on Intestinal Health and Lipid Metabolism in Dyslipidaemic Female Wistar Rats.” British Journal of Nutrition 119: 30–41. 10.1017/S0007114517003282. 29355095

[fsn372163-bib-0007] Becker, F. S. , C. Damiani , A. A. M. Melo , et al. 2014. “Incorporation of Buriti Endocarp Flour in Gluten‐Free Whole Cookies as Potential Source of Dietary Fiber.” Plant Foods for Human Nutrition 69: 344–350. 10.1007/s11130-014-0440-y. 25315266

[fsn372163-bib-0008] Benedé‐Ubieto, R. , O. Estévez‐Vázquez , P. Ramadori , F. J. Cubero , and Y. A. Nevzorova . 2020. “Guidelines and Considerations for Metabolic Tolerance Tests in Mice.” Diabetes, Metabolic Syndrome and Obesity: Targets and Therapy 13: 439–450. 10.2147/DMSO.S234665. 32110077 PMC7038777

[fsn372163-bib-0009] Bijkerk, C. J. , J. W. M. Muris , J. A. Knottnerus , A. W. Hoes , and N. J. de Wit . 2004. “Systematic Review: The Role of Different Types of Fibre in the Treatment of Irritable Bowel Syndrome.” Alimentary Pharmacology & Therapeutics 19: 245–251. 10.1111/j.0269-2813.2004.01862.x. 14984370

[fsn372163-bib-0010] Brazil. Instituto Nacional de Câncer (INCA) . 2022. How to Increase Fiber Intake in Your Diet. https://www.gov.br/inca/pt‐br/assuntos/causas‐e‐prevencao‐do‐cancer/dicas/alimentacao/como‐aumentar‐o‐consumo‐de‐fibras‐na‐sua‐alimentacao.

[fsn372163-bib-0011] Callahan, B. J. , P. J. McMurdie , M. J. Rosen , A. W. Han , A. J. A. Johnson , and S. P. Holmes . 2016. “DADA2: High‐Resolution Sample Inference From Illumina Amplicon Data.” Nature Methods 13: 581–583. 10.1038/nmeth.3869. 27214047 PMC4927377

[fsn372163-bib-0012] Cani, P. D. , R. Bibiloni , C. Knauf , et al. 2008. “Changes in Gut Microbiota Control Metabolic Endotoxemia‐Induced Inflammation in High‐Fat Diet–Induced Obesity and Diabetes in Mice.” Diabetes 57: 1470–1481. 10.2337/db07-1403. 18305141

[fsn372163-bib-0013] Casarotti, S. N. , S. S. Moura , M. S. Lima , et al. 2018. “Guava, Orange and Passion Fruit By‐Products: Characterization and Effect of Their Addition to Probiotic Yogurt.” Food Research International 106: 1–8. 10.1016/j.foodres.2018.01.046.29579892

[fsn372163-bib-0014] Chen, S. , Y. Zhou , Y. Chen , and J. Gu . 2018. “Fastp: An Ultra‐Fast All‐In‐One FASTQ Preprocessor.” Bioinformatics 34, no. 17: i884–i890. 10.1093/bioinformatics/bty560. 30423086 PMC6129281

[fsn372163-bib-0015] Cole, J. R. , B. Chai , R. J. Farris , et al. 2005. “The Ribosomal Database Project (RDP‐II): Sequences and Tools for High‐Throughput rRNA Analysis.” Nucleic Acids Research 33: D294–D296. 10.1093/nar/gki038. 15608200 PMC539992

[fsn372163-bib-0016] Didion, J. P. , M. Martin , and F. S. Collins . 2017. “Atropos: Specific, Sensitive, and Speedy Trimming of Sequencing Reads.” PeerJ 5: e3720. 10.7717/peerj.3720. 28875074 PMC5581536

[fsn372163-bib-0017] Dragano, N. R. V. , V. P. de Venancio , F. B. A. Paula , F. Della Lucia , M. J. de Oliveira , and L. Azevedo . 2010. “Influence of Marolo (*Annona crassiflora* Mart.) Pulp Intake on the Modulation of Mutagenic/Antimutagenic Processes and Its Action on Oxidative Stress in Vivo.” Plant Foods for Human Nutrition 65: 319–325. 10.1007/s11130-010-0191-3. 20878359

[fsn372163-bib-0018] Edgar, R. 2010. USEARCH. Lawrence Berkeley National Lab (LBNL).

[fsn372163-bib-0019] Föste, M. , C. Verheyen , M. Jekle , and T. Becker . 2020. “Fibres of Milling and Fruit Processing By‐Products in Gluten‐Free Bread Making: A Review of Hydration Properties, Dough Formation and Quality‐Improving Strategies.” Food Chemistry 306: 125451. 10.1016/j.foodchem.2019.125451. 31634767

[fsn372163-bib-0020] Gasmi, A. , M. Shanaida , O. Oleshchuk , et al. 2023. “Natural Ingredients to Improve Immunity.” Pharmaceuticals 16: 528. 10.3390/ph16040528. 37111285 PMC10143734

[fsn372163-bib-0021] Gaudier, E. , A. Jarry , H. M. Blottière , et al. 2004. “Butyrate Specifically Modulates MUC Gene Expression in Intestinal Epithelial Goblet Cells Deprived of Glucose. *American Journal of Physiology—Gastrointestinal and Liver* .” Physiology 287: G1168–G1174. 10.1152/ajpgi.00219.2004. 15308471

[fsn372163-bib-0022] Gibson, G. , R. Hutkins , M. E. Sanders , et al. 2017. “Expert Consensus Document: The International Scientific Association for Probiotics and Prebiotics (ISAPP) Consensus Statement on the Definition and Scope of Prebiotics.” Nature Reviews Gastroenterology & Hepatology 14: 491–502. 10.1038/nrgastro.2017.75. 28611480

[fsn372163-bib-0023] Gil‐Sánchez, I. , P. López‐Sánchez , A. González‐Sarrías , E. Gómez‐Plaza , J. C. Espín , and R. García‐Villalba . 2018. “Dynamic Gastrointestinal Digestion of Grape Pomace Extracts: Bioaccessible Phenolic Metabolites and Impact on Human Gut Microbiota.” Food Research International 107: 363–373. 10.1016/j.foodres.2018.02.022.

[fsn372163-bib-0024] Holscher, H. D. 2017. “Dietary Fiber and Prebiotics and the Gastrointestinal Microbiota.” Gut Microbes 8: 172–184. 10.1080/19490976.2017.1290756. 28165863 PMC5390821

[fsn372163-bib-0025] Hu, J. L. , S. P. Nie , F. F. Min , and M. Y. Xie . 2012. “Polysaccharide From Seeds of *Plantago asiatica* L. Increases Short‐Chain Fatty Acid Production and Fecal Moisture Along With Lowering pH in Mouse Colon.” Journal of Agricultural and Food Chemistry 60: 11525–11532. 10.1021/jf302169u. 23113806

[fsn372163-bib-0061] Jiang, H. , W. Zeng , X. Zhang , Y. Pei , H. Zhang , and Y. Li . 2022. “The Role of gut Microbiota in Patients With Benign and Malignant Brain Tumors: A Pilot Study.” Bioengineered 13, no. 3: 7847–7859. 10.1080/21655979.2022.2049959. 35291914 PMC9208447

[fsn372163-bib-0026] Koh, A. , F. De Vadder , P. Kovatcheva‐Datchary , and F. Bäckhed . 2016. “From Dietary Fiber to Host Physiology: Short‐Chain Fatty Acids as Key Bacterial Metabolites.” Cell 165: 1332–1345. 10.1016/j.cell.2016.05.041. 27259147

[fsn372163-bib-0027] Lima, M. D. C. , H. M. A. Nascimento , J. Y. P. Silva , J. L. B. Alves , and E. L. D. Souza . 2023. “Evidence for the Beneficial Effects of Brazilian Native Fruits and Their By‐Products on Human Intestinal Microbiota and Repercussions on Non‐Communicable Chronic Diseases—A Review.” Food 12: 3491. 10.3390/foods12183491. PMC10527964 37761200

[fsn372163-bib-0029] Magalhães, D. A. D. , W. T. Kume , F. S. Correia , et al. 2019. “High‐Fat Diet and Streptozotocin in the Induction of Type 2 Diabetes Mellitus: A New Proposal.” Anais da Academia Brasileira de Ciências 91: e20180314. 10.1590/0001-3765201920180314. 30916157

[fsn372163-bib-0030] Magne, F. , M. Gotteland , L. Gauthier , et al. 2020. “The Firmicutes/Bacteroidetes Ratio: A Relevant Marker of Gut Dysbiosis in Obese Patients?” Nutrients 12: 1474. 10.3390/nu12051474. 32438689 PMC7285218

[fsn372163-bib-0031] Magoč, T. , and S. L. Salzberg . 2011. “FLASH: Fast Length Adjustment of Short Reads to Improve Genome Assemblies.” Bioinformatics 27, no. 21: 2957–2963. 10.1093/bioinformatics/btr507. 21903629 PMC3198573

[fsn372163-bib-0032] Makki, K. , E. C. Deehan , J. Walter , and F. Bäckhed . 2018. “The Impact of Dietary Fiber on Gut Microbiota in Host Health and Disease.” Cell Host & Microbe 23: 705–715. 10.1016/j.chom.2018.05.012. 29902436

[fsn372163-bib-0033] McMurdie, P. J. , and S. Holmes . 2013. “Phyloseq: An R Package for Reproducible Interactive Analysis and Graphics of Microbiome Census Data.” PLoS One 8: e61217. 10.1371/journal.pone.0061217. 23630581 PMC3632530

[fsn372163-bib-0034] Mikryukov, V. 2022. metagMisc: Miscellaneous Functions for Metagenomic Analysis. R package version 0.0. 4. Computer Software. https://github.com/vmikk/metagMisc.

[fsn372163-bib-0035] Novelli, E. L. B. , Y. S. Diniz , C. M. Galhardi , et al. 2007. “Anthropometrical Parameters and Markers of Obesity in Rats.” Laboratory Animals 41: 111–119. 10.1258/002367707779399518. 17234057

[fsn372163-bib-0036] Oliveira, F. L. , T. Y. P. Arruda , M. C. Morzelle , A. P. A. Pereira , and S. N. Casarotti . 2022. “Fruit By‐Products as Potential Prebiotics and Promising Functional Ingredients to Produce Fermented Milk.” Food Research International 161: 111841. 10.1016/j.foodres.2022.111841. 36192971

[fsn372163-bib-0037] Oliveira, F. L. , M. C. Morzelle , M. M. S. Moretti , and S. N. Casarotti . 2023. “Fermentation of Araticum, Baru, and Pequi by‐Products by Probiotic Strains: Effects on Microorganisms, Short‐Chain Fatty Acids, and Bioactive Compounds.” Letters in Applied Microbiology 76: 912–923. 10.1093/lambio/ovad092. 37533204

[fsn372163-bib-0038] Pan, Y. , and F. Y. Jiao . 2024. “Link Between Childhood Obesity and Gut Microbiota.” World Journal of Gastroenterology 30: 3560–3563. 10.3748/wjg.v30.i30.3560. 39193569 PMC11346147

[fsn372163-bib-0039] Paone, P. , and P. D. Cani . 2020. “Mucus Barrier, Mucins and Gut Microbiota: The Expected Slimy Partners?” Gut 69: 2232–2243. 10.1136/gutjnl-2020-322260. 32917747 PMC7677487

[fsn372163-bib-0040] Paone, P. , F. Suriano , C. Jian , et al. 2022. “Prebiotic Oligofructose Protects Against High‐Fat Diet‐Induced Obesity by Changing the Gut Microbiota, Intestinal Mucus Production, Glycosylation and Secretion.” Gut Microbes 14: 2152307. 10.1080/19490976.2022.2152307. 36448728 PMC9715274

[fsn372163-bib-0060] R Core Team . 2021. R: A Language and Environment for Statistical Computing. R Foundation for Statistical Computing.

[fsn372163-bib-0041] Rau, S. , A. Gregg , S. Yaceczko , and B. Limketkai . 2024. “Prebiotics and Probiotics for Gastrointestinal Disorders.” Nutrients 16: 778. 10.3390/nu16060778. 38542689 PMC10975713

[fsn372163-bib-0042] Reynolds, A. N. , A. P. Akerman , and J. Mann . 2020. “Dietary Fibre and Whole Grains in Diabetes Management: Systematic Review and Meta‐Analyses.” PLoS Medicine 17, no. 3: e1003053. 10.1371/journal.pmed.1003053. 32142510 PMC7059907

[fsn372163-bib-0043] Roesler, R. , L. G. Malta , L. C. Carrasco , R. B. Holanda , C. A. S. Sousa , and G. M. Pastore . 2007. “Antioxidant Activity of Fruits From the Cerrado.” Food Science and Technology 27: 53–60. 10.1590/S0101-20612007000100010.

[fsn372163-bib-0044] Schliep, K. P. 2011. “Phangorn: Phylogenetic Analysis in R.” Bioinformatics 27: 592–593. 10.1093/bioinformatics/btq706. 21169378 PMC3035803

[fsn372163-bib-0045] Silva, M. R. , D. B. C. L. Lacerda , and A. S. Ferreira . 2008. “Chemical Characterization of Native Cerrado Fruits.” Ciência Rural 38: 1790–1793. 10.1590/S0103-84782008000600051.

[fsn372163-bib-0046] Slavin, J. 2013. “Fiber and Prebiotics: Mechanisms and Health Benefits.” Nutrients 5: 1417–1435. 10.3390/nu5041417. 23609775 PMC3705355

[fsn372163-bib-0047] Sun, C. Y. , Z. L. Zheng , C. W. Chen , B. W. Lu , and D. Liu . 2022. “Targeting Gut Microbiota With Natural Polysaccharides: Effective Interventions Against High‐Fat Diet‐Induced Metabolic Diseases.” Frontiers in Microbiology 13: 859206. 10.3389/fmicb.2022.859206. 35369480 PMC8965082

[fsn372163-bib-0048] Sun, Y. , S. Zhang , Q. Nie , et al. 2023. “Gut Firmicutes: Relationship With Dietary fiber and Role in Host Homeostasis.” Critical Reviews in Food Science and Nutrition 63: 12073–12088. 10.1080/10408398.2022.2098249. 35822206

[fsn372163-bib-0049] United Nations . 2024. “What Are the Sustainable Development Goals?” https://brasil.un.org/pt‐br/sdgs.

[fsn372163-bib-0050] Vacca, M. , G. Celano , F. M. Calabrese , P. Portincasa , M. Gobbetti , and M. De Angelis . 2020. “The Controversial Role of Human Gut Lachnospiraceae.” Microorganisms 8: 573. 10.3390/microorganisms8040573. 32326636 PMC7232163

[fsn372163-bib-0051] Willemsen, L. E. , M. A. Koetsier , S. J. H. van Deventer , and E. A. F. van Tol . 2003. “Short Chain Fatty Acids Stimulate Epithelial Mucin 2 Expression Through Differential Effects on Prostaglandin E(1) and E(2) Production by Intestinal Myofibroblasts.” Gut 52, no. 10: 1442–1447. 10.1136/gut.52.10.1442. 12970137 PMC1773837

[fsn372163-bib-0052] Winer, D. A. , H. Luck , S. Tsai , and S. Winer . 2016. “The Intestinal Immune System in Obesity and Insulin Resistance.” Cell Metabolism 23: 413–426. 10.1016/j.cmet.2016.01.003. 26853748

[fsn372163-bib-0053] Yadav, M. K. , I. Kumari , B. Singh , K. K. Sharma , and S. K. Tiwari . 2022. “Probiotics, Prebiotics and Synbiotics: Safe Options for Next‐Generation Therapeutics.” Applied Microbiology and Biotechnology 106: 505–521. 10.1007/s00253-021-11646-8. 35015145 PMC8749913

[fsn372163-bib-0054] Zhang, X. , J. Zhou , H. Li , X. Li , H. Yang , and X. Fan . 2024. “Gut Microbiota and Metabolic Health: Mechanisms and Therapeutic Perspectives.” Gut Microbes 18, no. 1: 2644677. 10.1080/19490976.2026.2644677.

